# Supplementary figures and images for: Mental development is associated with cortical connectivity of the ventral and nonspecific thalamus of preterm newborns
Source: Brain Behav. 2020 Aug 13;10(10):e01786. doi: 10.1002/brb3.1786 (PMC7559616; doi:10.1002/brb3.1786)

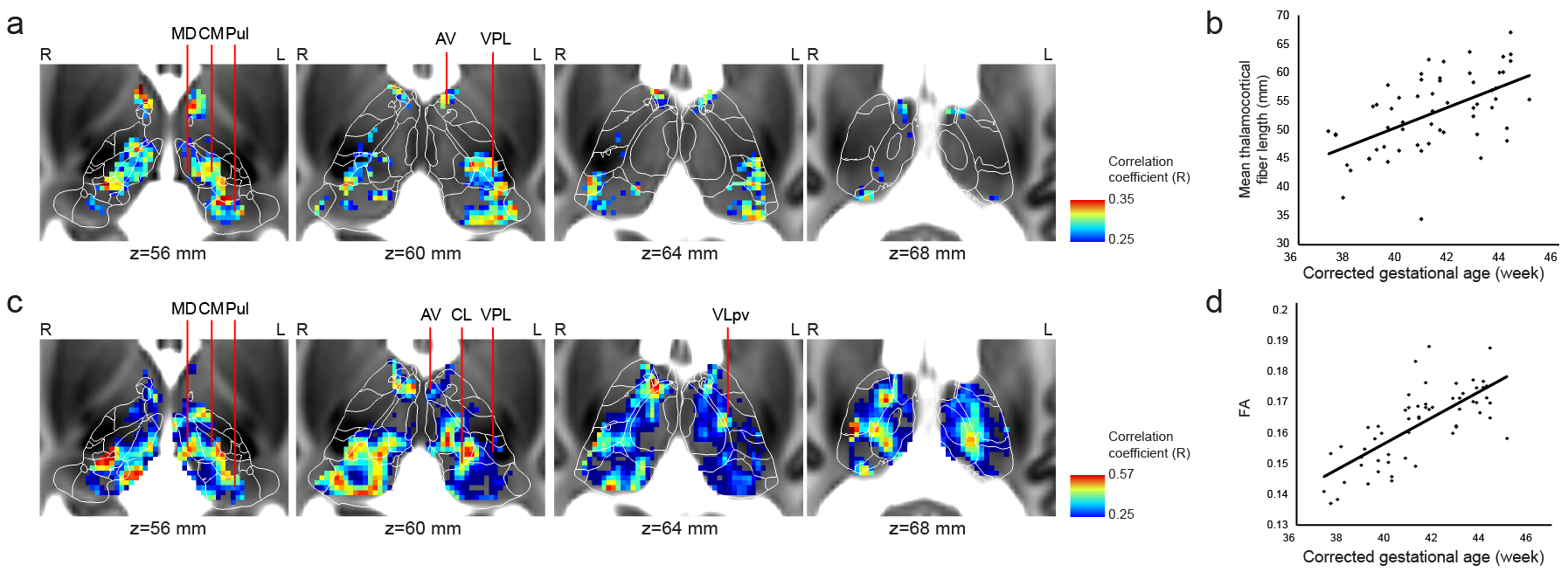

Supplement: Supplementary file 1 — Fig S1 [file BRB3-10-e01786-s001.tif]
