# Supplementary material for: Mental development is associated with cortical connectivity of the ventral and nonspecific thalamus of preterm newborns
Source: Brain Behav. 2020 Aug 13;10(10):e01786. doi: 10.1002/brb3.1786 (PMC7559616; doi:10.1002/brb3.1786)
Supplement: Supplementary file 2 — Table S1‐S3 [file BRB3-10-e01786-s002.docx]

**Supplementary Table 1. Post hoc stepwise linear regression analysis of the relationship between TC and MDI: all clinical and demographic variables.**

| Variable | Beta | t | p | Partial correlation | Entered into model |
| --- | --- | --- | --- | --- | --- |
| GA at birth | .220 | 1.859 | .069 | .257 | No |
| PMA at scanning | -.210 | -1.724 | .091 | -.239 | Yes |
| Birth weight | .200 | 1.623 | .111 | .226 | No |
| Birth weight, Z-Score | .151 | 1.218 | .229 | .171 | No |
| Head circumference | .169 | 1.379 | .174 | .193 | No |
| Head circumference, Z-Score | .131 | 1.054 | .297 | .149 | No |
| Parental SES | -.375 | -3.327 | .002 | -.429 | Yes |
| BPD grade | -.320 | -2.819 | .007 | -.374 | No |
| Duration of mechanical ventillation | -.405 | -3.760 | .000 | -.473 | Yes |
| Sepsis (Y/N) | -.278 | -2.378 | .021 | -.322 | No |
| EPO treatment group | .175 | 1.433 | .158 | .201 | No |
| Sex | -.061 | -.496 | .622 | -.071 | No |

**Supplementary Table 2. Thalamic nuclei abbreviations**

| AD | Anterodorsal nucleus | PuI | Inferior pulvinar |
| --- | --- | --- | --- |
| AM | Anteromedial nucleus | PuL | Lateral pulvinar |
| AV | Anteroventral nucleus | PuM | Medial pulvinar |
| CL | Central lateral nucleus | Pv | Paraventricular nuclei |
| CM | Centromedian nucleus | RN | Red nucleus |
| CeM | Central median nucleus | SG | Suprageniculate nucleus |
| Hb | Habenular nucleus | STh | Subthalamic nucleus |
| LD | Lateral dorsal nucleus | VAmc | Ventral anterior nucleus, magnocellular division |
| LGNmc | Lateral geniculate nucleus, magnocellular layers | VApc | Ventral anterior nucleus, parvocellular division |
| LGNpc | Lateral geniculate nucleus, parvocellular layers | VLa | Ventral lateral anterior nucleus |
| LP | Lateral posterior nucleus | VLpd | Ventral lateral posterior nucleus, dorsal division |
| Li | Limitans nucleus | VLpv | Ventral lateral posterior nucleus, ventral division (corresponds to VIM) |
| MDmc | Mediodorsal nucleus, magnocellcular division | VM | Ventral medial nucleus |
| MCpc | Mediodorsal nucleus, parvocellcular division | VPI | Ventral posterior inferior nucleus |
| MGN | Medial geniculate nucleus | VPLa | Ventral posterior lateral nucleus, anterior division |
| MV | Medioventral nucleus | VPLp | Ventral posterior lateral nucleus, posterior division |
| Pf | Parafascicular nucleus | VPM | Ventral posterior medial nucleus |
| Po | Posterior nucleus | mtt | Mamillothalamic tract |
| PuA | Anterior pulvinar | sPf | Subparafascicular nucleus |

**Supplementary Table 3. Post hoc stepwise linear regression analysis of the relationship between TC length and MDI: all clinical and demographic variables.**

| Variable | Beta | t | p | Partial correlation | Entered into model |
| --- | --- | --- | --- | --- | --- |
| GA at birth | .163 | 1.347 | .184 | .189 | No |
| PMA at scanning | -.184 | -1.504 | .139 | -.210 | Yes |
| Birth weight | .058 | .471 | .640 | .067 | No |
| Birth weight, Z-Score | .039 | .319 | .751 | .045 | No |
| Head circumference | .052 | .421 | .676 | .060 | No |
| Head circumference, Z-Score | .013 | .106 | .916 | .015 | No |
| Parental SES | -.367 | -3.198 | .002 | -.416 | Yes |
| BPD grade | -.267 | -2.234 | .030 | -.304 | No |
| Duration of mechanical ventillation | -.373 | -3.357 | .002 | -.432 | Yes |
| Sepsis (Y/N) | -.246 | -2.044 | .046 | -.280 | No |
| EPO treatment group | .188 | 1.546 | .129 | .216 | No |
| Sex | -.036 | -.294 | .770 | -.042 | No |
